# Dorsal intramedullary spinal epidermoid cysts: Report of two cases and review of literature

**DOI:** 10.4103/0019-5413.37005

**Published:** 2007

**Authors:** Rafael Cincu, Juan F Martin Lázaro, José Luis Capablo Liesa, José Ramón Ara Callizo

**Affiliations:** Department of Neurosurgery, Miguel Servet University Hospital, Zaragoza, Spain; * Department of Medical Biochemistry, Quiron Clinic, Madrid; ˆ Department of Neurology, Miguel Servet University Hospital, Zaragoza, Spain

**Keywords:** Cysts, epidermoid, intramedullary, magnetic resonance imaging

## Abstract

Intramedullary epidermoid cysts of the spinal cord are rare tumors, especially those not associated with spinal dysraphism. About 50 cases have been reported in the literature. Of these, only seven cases have had magnetic resonance imaging (MRI) studies. We report two cases of spinal intramedullary epidermoid cysts with MR imaging. Both were not associated with spina bifida. In one patient, the tumor was located at D4 vertebral level; while in the other, within the conus medullaris. The clinical features, MRI characteristics and surgical treatment of intramedullary epidermoid cyst are presented with relevant review of the literature.

Intraspinal epidermoid cysts are rare lesions and represent less than 1% of all intraspinal tumors in adults.^1^–^7^ We hereby present two cases of intramedullary epidermoid tumors.

## CASE REPORTS

### Case 1

**Figure 1:**
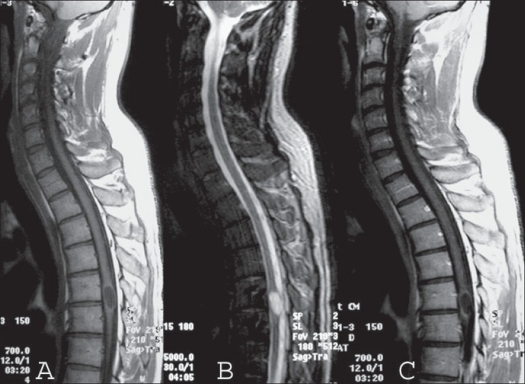
Mid sagittal T1 WI (A) of MRI of cervico dorsal spine shows a hypointense intramedullary lesion at D4 level, which became hyperintense on T2 WI (B) sequence with mild enhancement at periphery with gadolinium contrast (C)

**Figure 2:**
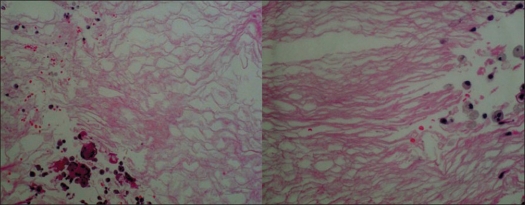
Histopathology showing stratified squamous epithelium containing degenerated squamous tissue

A 27-year-old gentleman presented with hypoesthesia in left lower limb and paresthesia in right lower limb for the past one year. He had lightning pain sensation in left lower limb for the last seven months. He had difficulty in gripping the right footwear for the last three months. The weakness continued to progress and included the whole right leg. There was no history of bowel/ bladder disturbances. Neurological examination revealed normal muscular tone; wasting of gastrocnemius muscle in right lower limb; power around hip joints, leg and foot of grade 4/5 in all muscle groups. Ankle and knee jerks were exaggerated in the right lower limb with extensor plantar response. Reflexes were normal in left lower limb. There was decreased propioception below T12 on the right side with 25% decrease in sensation to light touch on the left. On digital rectal examination, anal sphincter tone was normal. Upper limbs were normal. His general and systemic examination was normal. MRI dorsal spine revealed a well-defined intramedullary lesion about 18 mm in vertical diameter at the level of T5-T6, expanding the cord. The lesion was hypointense on T1 WI and hyperintense on T2 WI suggestive of cyst without perilesional edema. It showed mild peripheral enhancement after contrast administration and a diagnosis of astrocytoma was suspected [Figure 1]. The patient underwent T4 to T7 laminectomy. The dura was opened and a posterior mid line myelotomy was performed followed by a near-total excision of the lesion and duroplasty. Intra-operatively, there were white flaky fragments suggestive of epidermoid lesion. On histopathology the lesion had a thin fibrous capsule, encircled by gliotic tissue. Cyst was lined by compressed stratified squamous epithelium and contained degenerated squamous tissue [Figure 2]. All these features were characteristic of intramedullary epidermoid, with secondary inflammatory tissue. Immediately after the surgery, the patient worsened to grade 3/5 power in the right lower limb, which recovered over a period of next two weeks to the preoperative level.

### Case 2

A 28-year-old female presented with history of pain in the right thigh and frequent incontinence of urine for the past six years. She developed difficulty in gripping the footwear in both feet for the last three months. Clinical examination revealed power of grade 3/5 at the ankle joints. Sensory examination revealed 50% hypoesthesia below L4 dermatomes including perianal region for all modalities of sensation. Ankle jerks were absent bilaterally. Digital rectal examination revealed decreased anal sphincter tone. Upper limbs were normal. Other general and systemic examination was normal. MRI revealed a well-defined intramedullary lesion in the conus-epiconus region, which was expanding the cord. The lesion was hypointense on T1 WI and hyperintense on T2 WI. The patient underwent D12-L2 laminectomy. The conus region was markedly expanded; and through a midline myelotomy, near-total excision of the tumor including the capsule was performed. Histopathology confirmed the diagnosis of epidermoid cyst. She improved; and at four months followup, the ankle power was grade 4/5, minimal patchy sensory hypoesthesia and no urinary incontinence.

## DISCUSSION

Congenital epidermoid cysts of spinal cord are more common than acquired lesions.^8^ Congenital epidermoid cysts originate from displaced ectoderm inclusions arising in early fetal life and possibly may be associated with defective closure of the dural tube.^8^,^9^ Acquired epidermoid cysts have been found years after single or multiple lumbar spinal punctures and are thought to result from iatrogenic penetration of skin fragments.^8^,^10^,^11^ Thoracic region (between D4-D8 levels) is the favorite site of the intramedullary epidermoid cysts^6^,^9^,^12^ followed by the lumbar cord^12^; and rarely, these lesions involve cervical cord [Table 1].^1^ The diagnosis of intramedullary epidermoid cyst is often based on operative and histological finding.^2^ Magnetic resonance imaging (MRI) reduces the delay in diagnosis, and evidence has accumulated that these lesions may be preoperatively suspected.^9^,^12^,^13^ Epidermoid cysts are generally characterized on MRI by an important variability of signal intensity between the different cases and, at times, between the different parts of the same cysts; other features include the absence of edema in surrounding tissue, fairly well-defined limits and peripheral enhancement on injection of gadolinium.^1^,^2^,^6^,^7^,^12^,^13^ In both our cases the lesions were well defined and there was no surrounding edema. The disparity in signal intensity most likely reflects variable lipid and protein composition in these lesions. In addition it has been noticed that the margins of these lesions are ‘shaggy,’ possibly because of chronic inflammatory response to the squamous tissue ‘leak’ through the capsule and variable gliosis along the margin, extending into the cord. This feature may be of help in differentiating these lesions from other intramedullary tumours.^1^,^2^,^6^,^7^,^12^,^13^ Total resection of epidermoid cyst is the treatment of choice.^6^,^9^,^14^ However, when the capsule is intimately attached to the spinal cord or located within its confines, attempts to remove the cyst wall completely are unnecessary and carry a high risk of neurological deficit.^2^ In summary, MRI is the investigation of choice for intramedullary epidermoids, and surgical excision of these lesions results in the improvement of neurological functions [Table 1].

**Table 1:** Selected previous reports of intraspinal epidermoid tumors involving dorsal spinal cord

Author	Age/sex	Site	Presenting symptoms	Diagnostic modality	Management	Follow up
Kikuchi *et al*.^15^	44 year Male	Dorsal	Right leg numbness	MRI	Subtotal removal	Gait disturbance remained
Bretz *et al*.^16^	59-year Female	Cervico-dorsal	Spastic paraplegia of the lower limbs	MRI	Initially surgery, recurrence treated with radiotherapy	Had multiple recurrences
Chandra *et al*.^3^	18 year Female	Dorsal	Deep-seated pain in the left thigh	MRI	Surgery	Improved
			Progressive difficulty in walking			
	28 year Female	Conus medullaris	Pain in the right thigh Frequent incontinence of urine	MRI	Surgery	Improved
Zavanone *et al*.^14^	51 year Female	Cervico-dorsal	Pain and weakness	MRI	Surgery	Improved
Ferrara *et al*.^17^	13-year Female	Dorsal	Recurrent low urinary tract infections	MRI	Surgery	Improved
			Urinary frequency Nocturnal enuresis			
Vallé *et al*.^18^	21 year Male	Conus medullarls	Motor disturbances of left lower limb	Myelogram and a post-myelogram CT	Surgery	Improved
			More recent urinary incontinence			
Scarrow *et al*.^19^	31 year Female	Dorsal	Progressive lower extremity weakness Spasticity	MRI	Surgery	Improved
